# Rapid sample delivery for megahertz serial crystallography at X-ray FELs

**DOI:** 10.1107/S2052252518008369

**Published:** 2018-07-27

**Authors:** Max O. Wiedorn, Salah Awel, Andrew J. Morgan, Kartik Ayyer, Yaroslav Gevorkov, Holger Fleckenstein, Nils Roth, Luigi Adriano, Richard Bean, Kenneth R. Beyerlein, Joe Chen, Jesse Coe, Francisco Cruz-Mazo, Tomas Ekeberg, Rita Graceffa, Michael Heymann, Daniel A. Horke, Juraj Knoška, Valerio Mariani, Reza Nazari, Dominik Oberthür, Amit K. Samanta, Raymond G. Sierra, Claudiu A. Stan, Oleksandr Yefanov, Dimitrios Rompotis, Jonathan Correa, Benjamin Erk, Rolf Treusch, Joachim Schulz, Brenda G. Hogue, Alfonso M. Gañán-Calvo, Petra Fromme, Jochen Küpper, Andrei V. Rode, Saša Bajt, Richard A. Kirian, Henry N. Chapman

**Affiliations:** a Center for Free-Electron Laser Science, Deutsches Elektronen-Synchrotron DESY, Notkestrasse 85, 22607 Hamburg, Germany; b Department of Physics, Universität Hamburg, Luruper Chaussee 149, 22761 Hamburg, Germany; c The Hamburg Center for Ultrafast Imaging, Universität Hamburg, Luruper Chaussee 149, 22761 Hamburg, Germany; d Institute of Vision Systems, Hamburg University of Technology, Harburger Schlossstrasse 20, 21079 Hamburg, Germany; e Photon Science, DESY, Notkestrasse 85, 22607 Hamburg, Germany; f European XFEL GmbH, Holzkoppel 4, 22869 Schenefeld, Germany; g Arizona State University, 550 E. Tyler Drive, Tempe, AZ 85287, USA; h Universidad de Sevilla, Department of Aerospace Engineering and Fluid Mechanics, Camino de los Descubriemientos s/n, 41092 Sevilla, Spain; i Max Planck Institute of Biochemistry, Department of Cellular and Molecular Biophysics, 82152 Martinsried, Germany; j LCLS, SLAC National Accelerator Laboratory, 2575 Sand Hill Road, Menlo Park, CA 94025, USA; k Department of Physics, Rutgers University Newark, Newark, NJ 07102, USA; l Stanford PULSE Institute, SLAC National Accelerator Laboratory, 2575 Sand Hill Road, Menlo Park, CA 94025, USA; m Deutsches Elektronen-Synchrotron DESY, Notkestrasse 85, 22607 Hamburg, Germany; n Biodesign Institute, School of Life Sciences, Arizona State University, Tempe, AZ 85287, USA; o Laser Physics Centre, Research School of Physics and Engineering, Australian National University, Canberra, ACT 2601, Australia

**Keywords:** X-ray free-electron lasers, FELs, X-ray FEL pulse trains, megahertz repetition rates

## Abstract

Liquid microjets are a common means of delivering protein crystals to the focus of X-ray free-electron lasers (FELs) for serial femtosecond crystallography measurements. The high X-ray intensity in the focus initiates an explosion of the microjet and sample. With the advent of X-ray FELs with megahertz rates, the typical velocities of these jets must be increased significantly in order to replenish the damaged material in time for the subsequent measurement with the next X-ray pulse. This work reports the results of a megahertz serial diffraction experiment at the FLASH FEL facility using 4.3 nm radiation. The operation of gas-dynamic nozzles that produce liquid microjets with velocities greater than 80 m s^−1^ was demonstrated. Furthermore, this article provides optical images of X-ray-induced explosions together with Bragg diffraction from protein microcrystals exposed to trains of X-ray pulses repeating at rates of up to 4.5 MHz. The results indicate the feasibility for megahertz serial crystallography measurements with hard X-rays and give guidance for the design of such experiments.

## Abstract 2

Sample delivery is a major challenge to performing serial crystallography experiments at upcoming high-repetition-rate X-ray free-electron lasers. The feasibility of using gas-driven liquid jets for this purpose at the FLASH facility in Hamburg has been studied.

## 1. Introduction   

Serial femtosecond crystallography (SFX) uses focused X-ray pulses from free-electron laser (FEL) sources to record ‘snapshot’ diffraction patterns of individual macromolecular crystals (Chapman *et al.*, 2011 ▸; Boutet *et al.*, 2012 ▸). This method provides a new paradigm for protein-structure determination by recording many tens of thousands of patterns of such crystals that are then used to estimate crystal structure factors, from which a molecular structure can be derived. A long-standing problem in crystallography has been radiation damage, which limits the total exposure that a macromolecular crystal can tolerate (Henderson, 1995 ▸; Owen *et al.*, 2006 ▸). The use of X-ray FEL pulses allows the X-ray dose that the sample can tolerate to be increased thousands of times, as long as the pulse duration is short enough to freeze significant atomic motion induced by the X-ray interaction (Neutze *et al.*, 2000 ▸). Under such conditions, protein crystals even smaller than 1 µm^3^ can be used for structure determination, measurements can be made at room temperature, and time-resolved measurements of irreversible processes can be carried out by synchronizing the measurement (Barends *et al.*, 2015 ▸; Pande *et al.*, 2016 ▸; Stagno *et al.*, 2017 ▸; Kupitz *et al.*, 2016 ▸). Focusing an X-ray FEL pulse onto such a sample leads to its complete destruction, which is why only one diffraction pattern can be collected per microcrystal. Many SFX experiments, especially time-resolved studies and *de novo* phasing experiments, require the collection of a very large number of individual diffraction patterns to accurately measure small variations in signal levels (Barends *et al.*, 2015 ▸; Pande *et al.*, 2016 ▸; Nass *et al.*, 2016 ▸). Consequently, the total time required to collect a complete data set (under a particular experimental condition or time delay) has an inverse dependence on the rate at which patterns can be acquired, limited by the rate at which pulses are generated by the FEL. At the European XFEL, up to 27 000 pulses s^−1^ will be delivered to an experimental endstation, which is over 200 times more than the 120 Hz of the Linac Coherent Light Source (LCLS), where many serial femtosecond crystallography experiments have been conducted. Such a high-repetition rate would enable time-resolved measurements over many time-points to capture intermediate structures or carry out large combinatorial experiments such as fragment screening (Blundell, 2017 ▸; Beyerlein, Dierksmeyer *et al.*, 2017 ▸; Keedy *et al.*, 2017 ▸).

Meeting this capability requires the means to record diffraction patterns at a high rate, along with a means to deliver the sample across the beam with sufficiently high velocity. Although current pixel-array X-ray detector technologies cannot acquire 27 000 frames s^−1^, a fast electronic veto system could be used to store only measurements with useful diffraction (Trunk *et al.*, 2017 ▸). Furthermore, algorithms can be used to index patterns that consist of multiple crystals in random orientations (Gildea *et al.*, 2014 ▸; Ginn *et al.*, 2016 ▸; Beyerlein, White *et al.*, 2017 ▸) and thus could utilize frames exposed over multiple pulses. There are no such workarounds for delivering the sample too slowly. Achieving sample delivery that is fast enough is challenging since the European XFEL generates ten pulse trains per second, each lasting up to 600 µs with as little as 220 ns between pulses in the train, a rate of 4.5 MHz. For a stationary (non-scanned) X-ray beam, the sample must move far enough in 220 ns to clear any volume of sample that was affected by the previous pulse. The radius of the pre-exposed interaction volume also includes the low-intensity ‘wings’ of the focused X-ray beam. If the radius of this interaction region is of the order of 20 µm, samples have to travel faster than 100 ms^−1^. The interaction between a focused X-ray FEL pulse and the liquid jet, a common method to deliver microcrystals to the X-ray beam, usually results in a visible glow that can be observed in experiments, but it was not until short-exposure photographs were made moments after X-ray exposure that the dynamics and extent of the liquid jet explosion were appreciated (Stan *et al.*, 2016 ▸). These studies determined the width of the destruction, and hence the velocity of the jet required for a given pulse rate, and showed that thinner, faster jets would have the advantage of smaller volumes of destruction.

A promising delivery system to introduce protein crystals to the X-ray FEL beam at the required 4.5 MHz rate is a fast liquid microjet produced by gas-flow focusing using a so-called gas dynamic virtual nozzle (GDVN) (Gañán-Calvo, 1998 ▸; DePonte *et al.*, 2008 ▸; Beyerlein *et al.*, 2015 ▸). Such microjets were used for the very first serial femtosecond crystallography experiments at LCLS (Chapman *et al.*, 2011 ▸), keeping the crystals in their growth solution at room temperature, even when operated in vacuum. Such jets typically run at velocities of up to 10–20 m s^−1^ (Beyerlein *et al.*, 2015 ▸), which is fast enough for 120 Hz pulses, but is not sufficient for the 4.5 MHz rate of the European XFEL. The velocity of a liquid jet is equal to the volume-flow rate divided by the cross-sectional area of the jet. Increasing the jet velocity therefore requires increasing the flow rate without increasing the diameter, reducing the diameter without reducing the flow rate, or both. In a previous study, it was found that the jet velocity from a ceramic GDVN could be doubled from ∼12 to 25 m s^−1^ by increasing the gas flow from 6 to 35 mg min^−1^. Extrapolating this trend to velocities approaching 100 m s^−1^ demands a gas load too large for in-vacuum measurements, producing a very unstable gas flow and resulting in an unstable jet (Si *et al.*, 2009 ▸). Thus, a new nozzle design is needed.

In this paper, we report the results of experiments conducted at the soft-X-ray free-electron laser in Hamburg (FLASH) (Feldhaus, 2010 ▸) to test nozzle design principles that enable jet velocities needed for megahertz repetition rates. At the time of this study (before operation of the European XFEL) this was the only FEL with megahertz repetition rates where such studies could be conducted. Even though this FEL does not reach the photon energies required for protein crystallography, we could perform experiments with a similar dose to the jet and the sample as expected at the European XFEL. This was achieved with a suitable choice of photon energy (near the carbon absorption edge), fluid (ethanol) and tight focusing of the X-ray beam. Our experiments were designed to simulate some of the expected conditions at the European XFEL facility in order to determine the viability of megahertz SFX experiments, explore the interaction of thin liquid jets with intense X-ray pulses and to gain feedback on nozzle designs. In addition to the X-ray-induced gap in the liquid jet, shock waves have been observed during LCLS experiments by Stan *et al.* (2016 ▸) when using a very thick jet with a 20 µm diameter, but at the 120 Hz pulse rate of the LCLS experiments. The shocks dissipated before interacting with any crystals that arrived in the interaction region at the time of the next X-ray pulse. The effects of such a shockwave on crystal structures have not been observed previously. As a result of the low photon energies in our experiments (288 eV), we can only report the findings that low-resolution diffraction can be observed in subsequent pulses at 1 µs spacing, which is necessary, though insufficient, in determining the feasibility of SFX experiments at atomic resolution.

We give an overview of the explosive interaction of intense femtosecond X-ray pulses with jets in Section 2 of this article and present design criteria to achieve fast jets in Section 3. In Section 3, we further review the properties of liquid jets produced by GDVNs and their dependence on nozzle geometry, liquid properties and pressure in order to describe the changes in design needed to create fast jets. We present the details of the experiment at FLASH in Section 4 and the results in Section 5.

## 2. Interaction of intense X-ray pulses with liquid microjets   

The interaction between liquid microjets and intense X-ray laser pulses were studied previously by Stan *et al.* (2016 ▸) at the CXI endstation at the LCLS using 8.2 keV photons. The study exposed liquid microjets and microdrops to varying X-ray-pulse fluences and showed that even very attenuated X-ray pulses of 50 µJ µm^−2^ fluence resulted in a strong interaction with the microjet. As revealed through direct imaging, the energy deposited isochorically into a jet by an X-ray pulse leads to an explosion that produces a gap in the jet and possibly a pressure wave that propagates along the remaining jet. Stan *et al.* (2016 ▸) further determined that the gap size evolves in a sequence of stages, and the dynamics of each stage are determined by different physical mechanisms. Fortunately, for the case of experiments with low-viscosity microjets at high-repetition-rate XFELs, the relevant gap dynamics are limited to the first stage of growth, which is driven by the explosion and can be modeled analytically. Stan *et al.* (2016 ▸) found that for jet diameters *d*_jet_ of a few micrometres and an X-ray spot size matched to that diameter, the gap sizes Δ_gap_ at the end of stage I grew at a rate proportional to *d*_jet_. Furthermore, the dependence of this gap size on the X-ray energy absorbed in the jet per unit volume of jet material *U*_X-ray_ is: 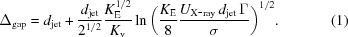
Here, *K*_E_ and *K*_v_ are empirical dimensionless constants found to be approximately 0.1 for the LCLS experiment, Γ = 0.5 is the Grüneisen coefficient for liquid water and σ is the surface tension of the jet medium. As the energy deposited per unit volume of the jet increases, the resulting gap also increases. This density can also be characterized by the dose to the jet, given by 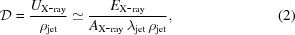
for a density ρ_jet_ of the jet medium, absorption length λ_jet_ of this same medium at the X-ray photon energy and a pulse of energy *E*_X-ray_ focused into an area *A*_X-ray_. The approximation to 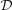 in equation (2) is the skin dose, which is approximately equal to the dose throughout the entire jet thickness when λ_jet_ ≫ *d*_jet_. At the low X-ray energies of our FLASH experiments, where λ_jet_ ≃ *d*_jet_, the absorbed energy decreases significantly along the X-ray path in the jet. For a jet of diameter 3.5 µm, typical for SFX experiments, Stan *et al.* (2016 ▸) found that the final gap caused by exposure to a 1 mJ µm^−2^ X-ray pulse with a photon energy of 8.2 keV after the evolution of all stages is ∼60 µm. This means that for the jet to recover, it has to move at least 30 µm (or half the gap size) before the next X-ray pulse arrives. This is not a problem at the LCLS, where the repetition rate of X-ray pulses is 120 Hz. In such a case, the jet only needs to move at a velocity of about 4 mm s^−1^, which even allows sample delivery using very slow extrusions of viscous media from nozzles (Weierstall *et al.*, 2014 ▸).

With the 220 ns inter-pulse spacings expected from the European XFEL, the gap growth is limited to stage I explosion dynamics, described by Stan *et al.* (2016 ▸). Equation (1) indicates that the jet diameter has the largest influence on the gap in the jet, and by extrapolating to the highest X-ray-pulse fluences (∼5 mJ µm^−2^), we arrive at an approximate but simple rule for predicting the worst-case gap size in an SFX experiment: the maximum expected gap size is approximately equal to ten jet diameters. Therefore, the jet needs to translate by approximately five jet diameters (half the gap size) between pulses. For a jet diameter of 3.5 µm and an interval between pulses of 220 ns, the required jet velocity is (5 × 3.5 µm)/(0.22 µs) = 80 m s^−1^, which is about 4–8 times faster than a typical GDVN used for SFX experiments. To operate jets with high X-ray-pulse repetition rates requires increasing the jet velocity, decreasing the jet gap or achieving both of these conditions. Decreasing the diameter to reduce the gap is most readily achieved by speeding up the jet, which also works in our favor. Beyerlein *et al.* (2015 ▸) found that a decrease in the liquid-flow rate or an increase in the focusing gas mass-flow rate creates a thinner and faster jet. Following these observations, we fabricated injection-molded nozzles that allow for increased jet speeds and reduced diameters by allowing for both reduced liquid-flow rates and increased gas loads (Vega *et al.*, 2010 ▸; Montanero *et al.*, 2011 ▸).

## 3. Fast liquid microjets   

There are several considerations in the design of nozzles to produce a stable liquid jet of a micrometre diameter with a velocity of about 100 m s^−1^. For these jet velocities, jet dynamics are dominated by the inertia of the liquid. To avoid global dripping, the jet driving force has to be higher than the surface tension. This balance is determined by the Weber number, defined for flow-focused jets as We = Δ*P*_g_ *d*_jet_/σ, where Δ*P*_g_ is the gas pressure drop through the GDVN exit orifice. The Weber number must be >1 for stable jets. Given the small jet diameters here, viscous forces have to be considered even for low-viscosity liquids like water. The effect of viscous forces relative to the surface tension of the liquid is expressed by the dimensionless capillary number of the jet, given by Ca = μ*v*_jet_/σ for a jet velocity *v*_jet_, viscosity μ, and surface tension σ. Capillary numbers that are too small cause disruption of the jet by capillary breakup too close to the nozzle exit to be suitable for typical diffraction experiments (Gañán-Calvo, 2008 ▸). A capillary number of about five is ideal. Typical sample-carrier liquids for SFX experiments, such as water, polyethylene glycol in water, or ethanol have relatively low viscosities that are equal to about 1–5 mPa and surface tensions in the range of about 20–70 mN m^−1^. For these liquids and the desired velocity of 100 m s^−1^, the capillary number ranges from about 1 to 10, as required. However, the jet is only stable if the driving gas flow is not so high as to introduce instabilities such as lateral whipping (Acero *et al.*, 2012 ▸). To avoid these whipping instabilities, the Weber number should be <25. Indeed, when We ≃ 25, the jet achieves the maximum possible length relative to its diameter before breaking into droplets. This condition can be related to the jet velocity by considering the first-order energy balance in flow focusing (Gañán-Calvo, 1998 ▸), 1/2ρ_jet_ *v* ^2^_jet_ = Δ*P*_g_. Using We = 25 gives the relationship between the jet diameter and its velocity at optimum conditions of 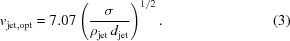
Because the liquid is practically incompressible, the jet diameter and the jet velocity are coupled by the liquid-flow rate. This flow rate has a minimum value (giving maximum velocity) that is dependent on the nozzle geometry, given as (Montanero *et al.*, 2011 ▸): 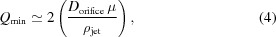
where *D*_orifice_ is the diameter of the exit orifice.

To achieve jet velocities where *v*_jet_ > 100 m s^−1^, and assuming the liquid is pure water, based on the considerations above, one requires jet diameters below 450 nm, pressures (Δ*P*_g_) above 5 MPa and liquid flow rates smaller than ∼1 µl min^−1^. Another consideration with regards to flow rate is the size of particles delivered to the X-ray beam and the rate of their delivery. Typically, suspensions of crystals or other particles in the carrier liquid do not account for more than 10% by volume. If we aim to obtain diffraction from a fraction *h* of the X-ray pulses (referred to as the hit fraction) that are arriving at a rate of *R*_X-ray_, we need the sample to flow at a rate of *Q* > *R*_X-ray_/(*c*_p_ *h*) for a mean particle volume of *v*_p_ and a concentration by volume of *c*_p_. For 1 µm^3^ crystals at 10% concentration by volume and a rate of *R*_X-ray_ = 4.5 MHz, a flow rate of at least 2.7 µl min^−1^ is required to reach a 100% hit fraction. If such a flow rate does not produce the necessary velocity to clear the X-ray beam in time for the next pulse, then the jet can be thinned and sped up, resulting in a lower hit fraction. In general, we see that the optimization of the jet properties must take into account various competing considerations, the most important of which are velocity and diameter. The discussion above suggests that existing nozzle designs should be modified to create thinner faster jets in order to optimize SFX measurements. We return to this optimization question in Section 5, where we consider whether or not it is warranted to reduce *R*_X-ray_, for example, to 1 MHz in order to maximize the hit rate *h*′ = *R*_X-ray_.

**Figure 1:**
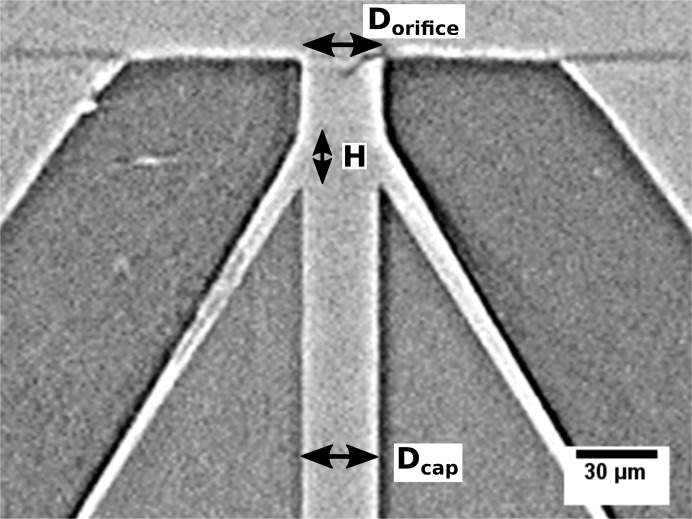
A slice through an X-ray tomogram of the nozzle used in the FLASH experiments. The nozzle is almost cylindrical/symmetric and consists of a sharpened glass capillary with an inner bore diameter of 30 µm surrounded by an injection-molded ceramic conical piece. The capillary transports the sample liquid to the nozzle exit where a free-standing liquid jet is formed by gas flowing through the interstitial space and out through the orifice. Tomography was performed with 25 keV photon energy at the P05 endstation at the PETRA III synchrotron facility.

Previous studies and experimental experience have shown that stable jets are produced with a nozzle design following the relationship of *D*_cap_ ≃ *D*_orifice_, where *D*_cap_ is the inner diameter of the liquid feeding capillary, as shown in Fig. 1 ▸. Furthermore, thin jets require the distance between the tip of this capillary and the gas orifice *H* to be small (Gañán-Calvo, 2008 ▸). With this geometry, the gas flow focuses and stabilizes the meniscus formed at the liquid capillary exit, and produces sustained jetting at low liquid-flow rates (Vega *et al.*, 2010 ▸). The minimum flow rate needed for a stable jet decreases with the inner diameter of the liquid-feeding capillary, independent of the applied gas flow. This reduces the meniscus volume that needs to be maintained by the flow, thereby reducing the required liquid-flow rate. We chose to make nozzles with dimensions of *D*_cap_ ≃ *D*_orifice_ ≃ 30 µm, which are smaller than the typical 50 µm diameters used for SFX, but that can be manufactured relatively easily (see Fig. 1 ▸ and the methods in Section 4).

## 4. Methods   

### 4.1. Nozzle manufacture   

For this experiment, GDVNs were made from ceramic injection-molded nozzle bodies with a 30 µm exit orifice (DePonte *et al.*, 2008 ▸; Beyerlein *et al.*, 2015 ▸). The wide opening on the entrance orifice of the body accepted two capillaries (Molex TSP030375 and TSP100375), one for supplying liquid and the other for gas. The sample feeding line, with a 30 µm inner diameter, was sharpened to a conical tip using a ULTRAPOL Fiberlab polisher (Ultra Tec) before inserting it into the ceramic piece. The channel in the ceramic piece leading to the nozzle tip had a square inner profile which guaranteed centering of the liquid capillary to the exit gas orifice. The conical tip also engaged with the matching inner cone of the ceramic piece to place the capillary at a defined distance from the exit orifice. This distance could be controlled in the assembly by polishing a slightly different cone angle onto the sample capillary. The second capillary, which feeds the He gas, was inserted into the body entrance next to the sample capillary. The entrance of the nozzle body was then sealed shut with a drop of epoxy. These nozzles form stable jets with liquid flow rates of ∼2–4 µl min^−1^ and gas mass flow rates between 3 and 25 mg min^−1^. They emit jets of up to 80 m s^−1^ with a diameter of 2.9 µm, as described below.

### 4.2. Samples   

Three samples were used in our soft-X-ray FEL experiments: pure water, pure ethanol and a protein nanocrystal suspension in an aqueous buffer. Ethanol was used because its absorption length at a photon energy of 288 eV, just greater than the carbon *K*-shell absorption edge, is only λ_jet_ = 0.45 µm (Henke *et al.*, 1993 ▸). Thus, essentially the full incident X-ray fluence is absorbed in the jet, yielding a higher dose than achievable with water [with λ_jet_ = 2.04 µm (Henke *et al.*, 1993 ▸)]. The crystal suspension consisted of photosystem I (PS I), a protein in the light-harvesting complex of the thermo­philic cyanobacterium *Thermosynechococcus elongatus* (Fromme & Witt, 1998 ▸). Crystallization was performed by desalting eluted PS I to <2 m*M* magnesium sulfate and concentrating to >300 µ*M* PS I using an Amicon 400 ml stirred cell-filtration system (EMD Millipore, USA) with a 100 kDa cutoff filter. Nanocrystals were then gently washed off the filter into the suspension in a quenching buffer containing 5 m*M* MES (pH 6.4), 0.02%(*w*/*v*) *N*-dodecyl-β-maltoside and stored in the dark at 4°C.

The crystallinity of the PS I sample was confirmed using second-harmonic generation spectroscopy (SONICC, Formulatrix, USA) in tandem with UV microscopy to confirm protein content. The mean size and the size distribution of the crystals were characterized using dynamic light scattering (DLS) (Spectro Size 302, Molecular Dimensions, UK) and nanoparticle tracking analysis (NTA) (NanoSight, Malvern, UK) as particles were too small for conventional microscopy. The DLS had to be modified to use a three-hanging-drop setup (1:9 dilution with quenching buffer) with 785 nm wavelength light and ten 20 s intervals used for the auto-correlation. The NTA measurements were performed after optimizing particle density to about 10^8^ crystals ml^−1^ (a 1000-fold dilution) using 18 MΩ water as a diluent. Size measurements were highly comparable with DLS measurements, showing a crystal size distribution of 395 ± 31 nm and NTA showing a mean size of 302 ± 17 nm with 80% of all particles between 155 and 469 nm. The NTA also provided an approximate density of 3.4 × 10^11^ crystals ml^−1^ for the undiluted solution.

### 4.3. Soft X-ray FEL experiments   

Experiments were conducted under vacuum at the FLASH facility using pulses of 288 eV photon energy (wavelength of 4.3 nm) and a duration of 100 ± 30 fs that were focused onto the liquid jet. The soft-X-ray-induced explosion of the liquid jet was observed by optical microscopy with short-pulse optical laser illumination (DILAS, 635 nm, 10 W, 10 ns modulated) synchronized to the X-ray pulses. Coherent X-ray diffraction from the jet and crystals in the jet were recorded in the forward direction using a PI-MTE X-ray detector (Princeton Instruments). Measurements were performed using single X-ray pulses, pulse trains of up to 250 X-ray pulses per train with an inter-pulse spacing of 1 µs and pulse pairs separated by 220 ns. The pulse sequence in the latter case was created by injecting a pair of electron bunches into RF buckets of the accelerator with an integer multiple of the 1.3 GHz bucket rate (Vogt *et al.*, 2017 ▸). The experimental results reported in this paper were obtained using two different experimental setups at different times. In one case, the CAMP end-station at FLASH (Strüder *et al.*, 2010 ▸) was used, in which the X-ray pulses were focused onto the jet by a pair of Kirkpatrick–Baez mirrors to a focal area of 6 × 8 µm. Accounting for transmission losses in the beamline and optics, the pulse energy in the focus was 2.8 ± 1.6 µJ or (6.1 ± 3.4) × 10^10^ photons, giving a fluence of 0.057 ± 0.034 µJ µm^−2^. The errors are derived from the measured jitter of FEL pulse energy. In the second setup, using our so-called ‘Bauhaus’ end station, the beam was focused to a spot of 1.3 × 1.3 µm, using an off-axis parabola operating at about 10° from normal incidence and coated with a reflective Sc/B_4_C/Cr multilayer film (Leontowich *et al.*, 2013 ▸). With a reflectivity at a 4.3 nm wavelength of 8.2%, the fluence achieved in this case was about ten times higher than the first setup at 0.5 ± 0.3 µJ µm^−2^.

**Figure 2:**
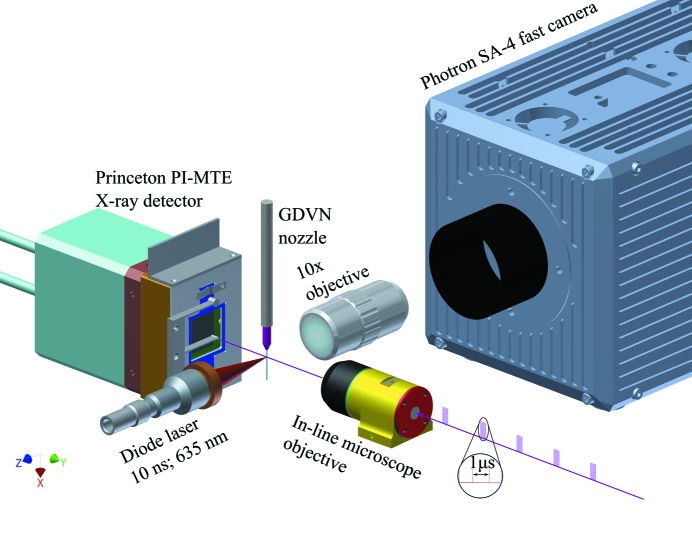
Schematic of the experimental setup in the Bauhaus chamber at FLASH. The X-ray-pulse trains pass through the in-line microscope into the interaction region. The nozzle is positioned so that the emerging jet intercepts the X-rays. The diffraction signal generated by the interaction between the jet and the X-ray beam is recorded by the Princeton PI-MTE detector and the effects of this interaction on the jet are monitored with a bright field microscope setup. Here, pulses from a fiber-coupled diode laser (DILAS) illuminate the jet and the image is formed with an in-vacuum microscope objective and a fast camera (Photron SA-4) located outside the vacuum chamber.

In both setups, two optical microscopes were used to monitor the liquid jet produced by the GDVN (see Fig. 2 ▸). The first microscope was collinear with the X-ray beam to provide an on-axis view for the purpose of jet alignment. The image was formed by a 10× Mitutoyo long-working-distance microscope objective with a central hole to allow the X-ray beam to pass through. A mirror mounted at 45°, also with a hole, transferred the image to a video camera. The second microscope gave a side view of the liquid jet, perpendicular to the X-ray beam in the horizontal plane. This microscope also used a 10× Mitutoyo long-working-distance microscope objective, but the image was recorded with a high-quality high-speed camera (Photron SA-4) located outside of the vacuum chamber. The side view microscope used bright-field illumination from a 10 ns pulsed diode laser to allow jet images to be recorded with high temporal and spatial resolution. The focal planes of both microscopes were centered at the X-ray focus and maintained in position to allow for fast and accurate jet positioning.

We recorded liquid jet optical images for a range of time delays after the start of the X-ray-pulse sequence (or single pulse). The zero time delay was established by reducing the delay until we observed no visual evidence of X-ray inter-action in the image. We collected one image per X-ray-pulse train at a FLASH inter-train repetition rate of 10 Hz. The time-delay sequence was cycled such that each delay was measured multiple times over the course of several hours in order to avoid bias caused by systematic drift in experimental parameters such as FEL pulse energy and liquid jet position. The position of the nozzle tended to vibrate by a few micrometres. We therefore digitally corrected our images for shift and in-plane rotation based on a cross-correlation analysis of the jet images. Since we cannot be certain of the relative position of the X-ray focus for each image, it was necessary to manually select images that showed the strongest X-ray interaction for each delay. The measured delays range from 0 to 2 µs for a single X-ray pulse, with an increased sampling of time delays below 100 ns in order to probe the fast dynamics during the onset of the jet explosion. We further measured time delays of up to 230 µs for the MHz pulse trains. The jet gaps induced by the X-rays were measured manually from the recorded images using the *ImageJ* program (Schneider *et al.*, 2012 ▸). The center–center distance between two different X-ray-induced gaps in the same frame was used to estimate the jet velocities.

**Figure 3:**
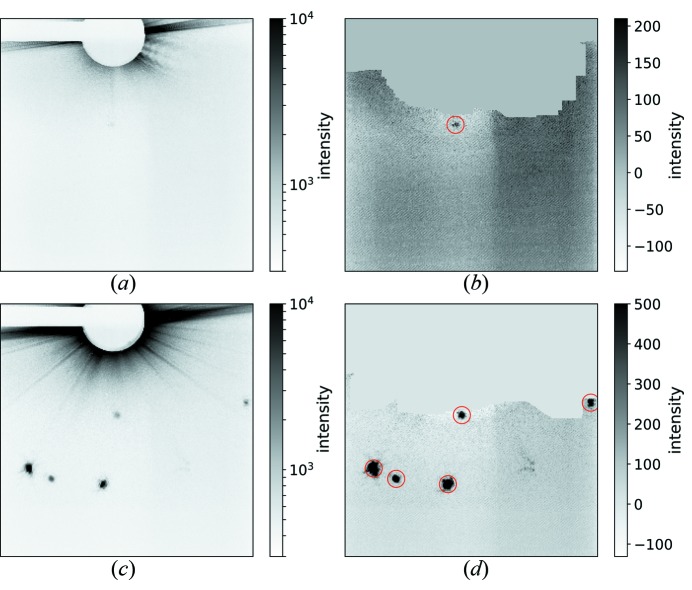
Example diffraction patterns recorded with one pulse (*a*) and (*b*), and 20 pulses (*c*) and (*d*), each at a fluence of 0.5 ± 0.3 µJ µm^−2^. Panels (*a*) and (*c*) display the raw images on a logarithmic gray scale and (*b*) and (*d*) show the corrected images on a linear gray scale after background subtraction, masking of jet streak, and identification of the peaks. The peak locations are indicated by red circles. Panels (*a*) and (*b*) show a typical pattern with one very weak peak whereas (*c*) and (*d*) show a strong pattern with multiple peaks from different crystals recorded in the course of the pulse train. One peak from the 100 class of Bragg reflections and multiple peaks from the 110 class were found. Based on the angle between some of the 110-peaks and the beam center, these cannot originate from the same crystal lattice.

Diffraction data were recorded separately to the optical images since the optical laser produced a strong background on the diffraction camera. The diffraction camera read-out time was about 1 s, so patterns were either recorded with single X-ray pulses or by integrating over a pulse train. We performed analysis of the soft-X-ray diffraction patterns of the PS I crystals to identify diffraction hits that showed clear evidence of Bragg diffraction and to determine how the number of hits scaled with the number of FEL pulses accumulated in each exposure. The diffraction patterns contained very strong streaks caused by diffraction from the liquid column. When a crystal was hit approximately one Bragg peak was formed. Fig. 3 ▸ shows a typical pattern and a very strong pattern. The first step in the processing pipeline was to identify and subtract the azimuthally symmetric background in the presence of Bragg peaks and jet diffraction. The background was estimated by calculating a *k*th order statistic in annular shells of the pattern, each corresponding to a particular scattering angle and resolution. We assumed the background in each particular annular resolution shell to be equal to the value associated with the index *k* = 0.1*N* in the sorted array of intensities in that shell, where *N* is the total number of pixel values in the shell. A relatively low *k* value was selected because of the large number of high-intensity values associated with the jet streak which cause background levels to be overestimated when using median (*k* = 0.5*N*) or mean values. After background subtraction, the jet-streak artifacts were masked with an algorithm that identified radial streaks of connected high-intensity pixels. Bragg reflections were identified with an algorithm that seeks regions of connected pixels that lie well above the local-background level.

## 5. Results and discussion   

**Figure 4:**
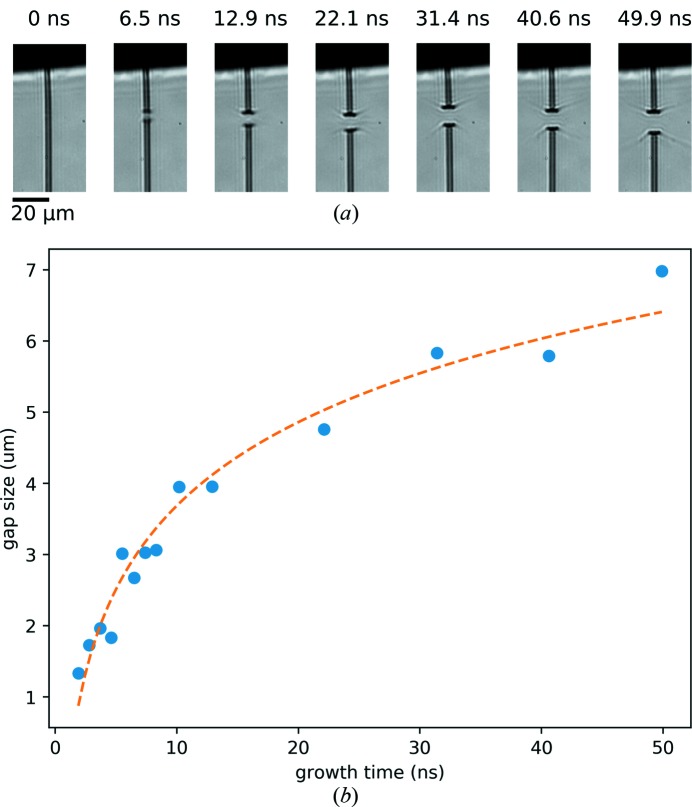
(*a*) Sideview images of a water jet at various delays after interception by a FLASH FEL pulse. (*b*) Plot of the evolution of the gap size in the first 50 ns after the FLASH FEL pulse hit the jet (solid circles) and the fit of a logarithmic function to the data (dashed line). The jet was flowing at a rate of 6.7 µl min^−1^ (helium mass-flow rate *Q*_g_ = 2.6 mg min^−1^) with a diameter *d*_jet_ = 3.1 µm and velocity *v*_jet_ = 60 m s^−1^. The dose deposited into the jet was approximately 30 MGy. Note that the position of the gap in the jet varies as a result of nozzle vibrations; the frames shown here are among those with the largest jet gaps recorded. The scale bar in the first panel of (*a*) is 20 µm and X-rays are incident from the left.

**Figure 5:**
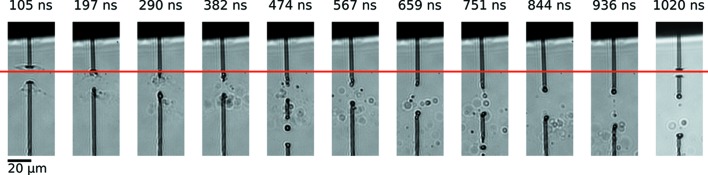
Sideview images showing the arrival of the second X-ray pulse in a train of pulses. The water jet was operated under the same conditions described in Fig. 4 ▸. The horizontal red line indicates that the jet has recovered and is stable in the X-ray interaction region after <290 ns. The last image in the sequence shows the jet after being hit by the second pulse in the pulse train. The scale bar is 20 µm. X-rays are incident from the left.

Using the CAMP end-station to deliver pulses of 0.057 ± 0.034 µJ µm^−2^ fluence in the 6 × 8 µm focus, the dose to the water jet was 28 ± 17 MGy and that to the ethanol jet was 160 ± 90 MGy. Here, the standard deviations correspond to the jitter in the FEL pulse energy. This dose is more than an order of magnitude higher than the enthalpy of vaporization for water (2.44 MJ kg^−1^), and thus, explosive boiling of the jet can be expected. Our choice of optical images showing the strongest interactions, made to overcome jitter in the beam and jet positions, may tend to experience higher doses than the mean values. Images showing the early evolution of the gap in a water jet after exposure to the initial X-ray pulse in the 1 MHz trains are displayed in Fig. 4 ▸(*a*). The jet had a diameter *d*_jet_ = 3.1 µm and velocity *v*_jet_ = 60 m s^−1^, flowing at *Q* = 6.7 µl min^−1^. A plot of the largest gap size observed at each delay, derived from such images, is shown in Fig. 4 ▸(*b*). The fit of a logarithmic function to the data, shown as the dashed line in Fig. 4 ▸(*b*), validates the applicability of the model of gap growth in stage I found by Stan *et al.* (2016 ▸). Fig. 5 ▸ shows the evolution over a much larger time range to 1.02 µs after the first X-ray pulse, which includes one frame measured 20 ns after the second pulse. These images reveal that the jet recovers in time for the arrival of the next pulse at a 1 MHz rate, but this relatively slow and thick jet would be too slow for experiments with the maximum 4.5 MHz rate anticipated at the European XFEL. From the horizontal red line in Fig. 5 ▸, it can be seen that the recovery time is <290 ns, and thus a higher velocity or smaller diameter are needed to make this jet suitable for experiments at repetition rates higher than ∼3.5 MHz for this particular dose.

**Figure 6:**
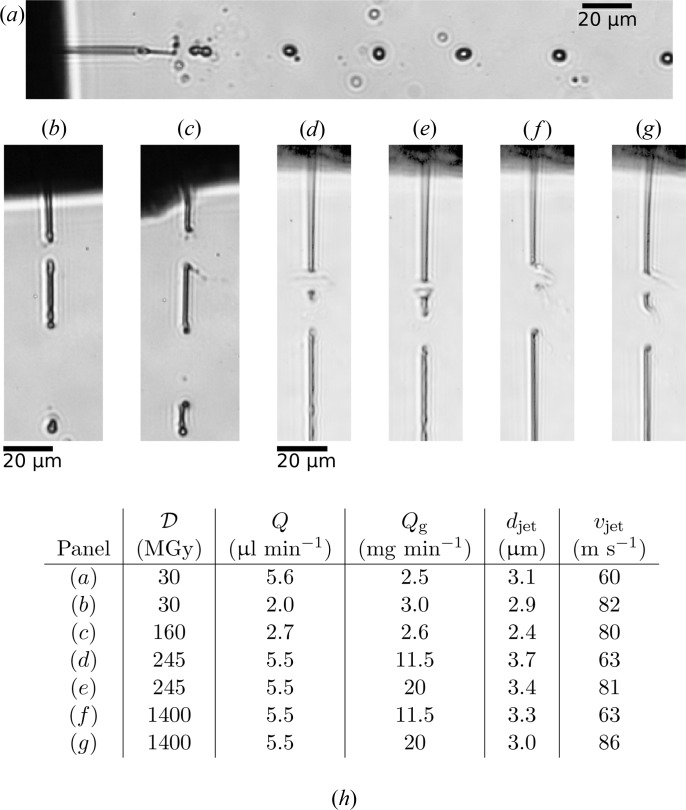
Comparison of the effects of X-ray-pulse structure and dose on different jets. (*a*) A water jet similar to the one shown in Figs. 4 ▸ and 5 ▸, exposed to a full train of 250 X-ray pulses at a repetition rate of 1 MHz, measured 90.1 s; after the initial pulse. (*b*) A thinner and faster water jet exposed to the same pulse train structure, measured 3.14 s after the initial pulse. (*c*) An ethanol jet also exposed to the same pulse train structure and measured at the same delay. (*d*) A slow water jet exposed to two FLASH pulses with a spacing of 221.5 ns. (*e*) A faster water jet exposed to the double-pulse, measured 50 ns after the second pulse. (*f*) A slow ethanol jet that barely recovers before the second X-ray pulse hits it 221.5 ns later, as measured 50 ns after the second pulse. (*g*) A fast ethanol jet under the same conditions. (*h*) A table with the experimental conditions of the jets shown in panels (*a*)–(*g*). The jets shown in (*a*)–(*c*) were formed with one particular nozzle and (*d*)–(*g*) were formed with another. A slice through an X-ray tomogram of the latter nozzle is shown in Fig. 1 ▸. The ethanol jets, especially (*f*) and (*g*), exhibit a non-symmetric gap formation where the jet explosion is directed towards the right side. The X-ray beam is incident from the left and is strongly absorbed at the surface of the jet. The energy deposition into the jet is consequently non-uniform.

Fig. 6 ▸(*a*) shows the effect of a full train of 250 pulses with 1 pulse spacing on a water jet with a velocity of 60 m s^−1^. Equally spaced drops are the result of repeated exposure to periodic X-ray pulses. By increasing the jet velocity to 82 m s^−1^ and decreasing the diameter to 2.9 µm, we increased the distance between the FEL-induced gaps and reduced the recovery time. Fig. 6 ▸(*b*) shows this faster jet 140 ns after the most recent pulse of the pulse train interaction with the jet. The dose to the water jet in these measurements, at about 30 MGy, was lower than usual dose of about 1 GGy experienced in SFX experiments at the CXI beamline at the LCLS. We approached this regime by changing the liquid from water to ethanol, which increased the dose to 160 MGy in the CAMP setup. For a similar jet velocity and diameter, a larger gap was observed as a result, shown by the comparison of Fig. 6 ▸(*c*) with the water jet image in Fig. 6 ▸(*b*), both recorded 140 ns after the most recent X-ray pulse. The ethanol jet was operated at a velocity of 80 m s^−1^ and with a 2.4 µm diameter. Even though the gap was larger, the jet still recovered before the next FEL pulse arrived. The downstream gap caused by the previous X-ray pulse was larger for water than for ethanol because of the high surface tension of water, which caused the free-standing jet segments to contract into spherical droplets more quickly.

With a 1.3 µm focus in the Bauhaus setup, giving pulse fluences of 0.5 ± 0.3 µJ µm^−2^, the dose to the water jet was 245 MGy and the dose to the ethanol jet was 1.4 GGy. Not only did we increase the dose, but we also decreased the pulse spacing to 221.5 ns, matching the shortest pulse interval at the European XFEL. Figs. 6 ▸(*d*) and 6 ▸(*e*) show slow and fast water jets, respectively, and Figs. 6 ▸(*f*) and 6 ▸(*g*) show slow and fast ethanol jets, respectively. The four images were all taken 50 ns after the second X-ray pulse; all four jets recovered in time before the second pulse arrived, suggesting that ∼3 µm jets may be compatible with 4.5 MHz SFX measurements.

Given our results so far, we now consider how to maximize the crystal hit rate with consideration of the interconnected experimental conditions. Since the crystals and the X-ray beam are probably smaller than the jet diameter of *d*_jet_ ≃ 3 µm, a simple geometric argument gives the approximate hit rate *h*′ ≃ *n*_p_ *d*_jet_ *A*_int_ *R*_X-ray_, where *n*_p_ is the particle number density and *A*_int_ is the interaction area, within which a crystal must be located in order to produce useful diffraction. To a good approximation, the jet gap given in equation (1) scales in direct proportion with the jet diameter, and since the jet velocity is constrained by *v*_jet_ ∝ *Q*/*d* ^2^_jet_, the maximum desired X-ray repetition rate is *R*_X-ray, max_ ≃ *v*_jet_/Δ_gap_ ∝ *Q*/*d* ^3^_jet_ and the corresponding maximum hit rate is *h*′_max_ ∝ *n*_p_ *A*_int_ *Q*/*d* ^2^_jet_ ∝ *n*_p_ *A*_int_ *v*_jet_. Since *n*_p_ and *A*_int_ are constrained by sample and beam characteristics, we have an effective scaling of *h*′_max_ 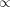 *v*_jet_ and thus a rather simple rule of thumb: for a given flow rate, hit rates are maximized by making jets as fast as possible. Of course, the jet velocity should not be faster than necessary to utilize the maximum X-ray repetition rate, and the highly sample-dependent jet-stability limitations may necessitate a reduction of the X-ray repetition rate. Most importantly, this all assumes that the jet explosions do not damage crystals located just upstream of the jet gap, which we discuss next.

Using the Bauhaus setup, we studied the potential influence of the explosion-induced pressure wave reported by Stan *et al.* (2016 ▸) on protein crystal diffraction. Secondary-radiation damage effects caused by FEL-induced radicals can be neglected, since typical diffusion coefficients (*D*) for these radicals range from 5.1 × 10^−10^ m^2^ s^−1^ in cases of hydroxyl radicals, to 4.9 × 10^−9^ m^2^ s^−1^ in cases of solvated electrons (Okuda *et al.*, 2009 ▸; Schmidt, Han & Bartels, 1992 ▸). The resulting diffusion length [*x* = 2(*Dt*)^1/2^] for electron diffusion in time *t* = 1 µs is ∼140 nm. We chose PS I membrane protein crystals for this study because their high solvent content (73%) and relatively weak contacts make them very soft and fragile. Furthermore, PS I crystals can be grown in small sizes that are comparable with the attenuation length of a protein, which is less than 1 µm at a 4.3 nm wavelength. Using a thin and fast jet (*d*_jet_ = 3.0 µm, *v*_jet_ = 70.0 m s^−1^ with *Q* = 5.7 µl min^−1^) to ensure a jet recovery time of less than 221.5 ns, we collected PS I diffraction data with 1, 2, 5, 10 and 20 X-ray pulses per pulse train. The limitations of the experimental operation mode for FLASH meant that only two pulse data were collected, with an inter-pulse spacing of 221.5 ns and an X-ray fluence of ∼0.85 µJ µm^−2^, whereas for 5, 10 and 20 pulses, an inter-pulse spacing of 1 µs was used with an X-ray fluence of about ∼0.5 µJ µm^−2^.

**Figure 7:**
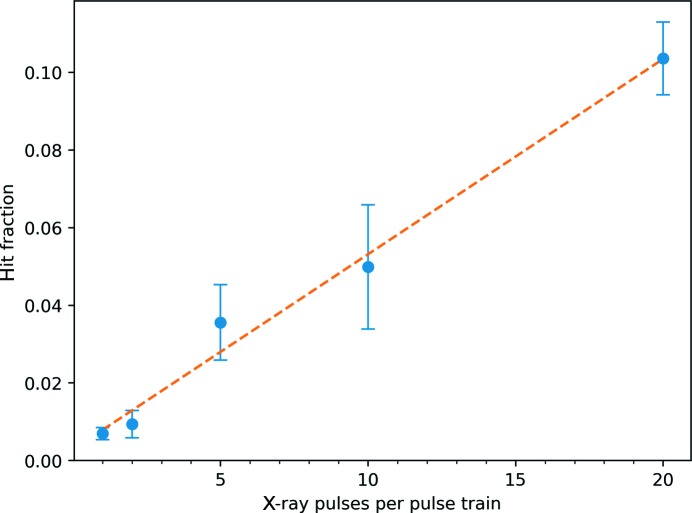
Hit fraction for different numbers of X-ray pulses in each pulse train. The error bars are derived from the standard deviation of hit fractions from many data collection runs under similar conditions. The dashed line is a linear fit.

**Figure 8:**
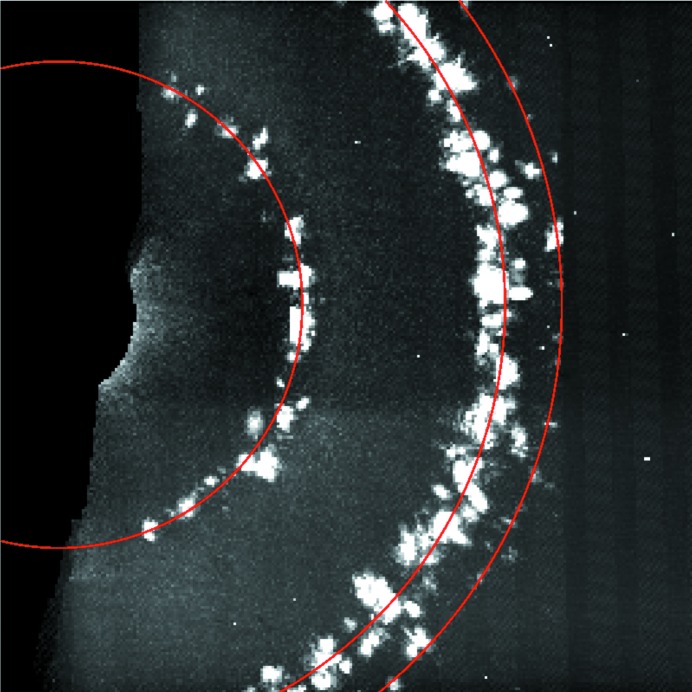
Virtual powder pattern from one run during the experiment. Photosystem I crystals have a hexagonal unit cell with *a* = *b* = 281 and *c* = 165.2 Å, space group *P*6_3_. The innermost ring is comprised of 100-type reflections at a resolution of ∼41 µm^−1^, the intermediate ring contains nearly overlapping 110- and 101-type reflections at resolutions ≃ 72 µm^−1^ and the outermost ring consists of 200-type reflections at ∼82 µm^−1^.

Fig. 7 ▸ shows a plot of the average number of crystal lattices recorded per diffraction pattern, accumulated over the pulse train as a function of the number of pulses per train. It reveals that the average number of crystal hits increases linearly with the number of X-ray pulses that were accumulated, indicating that the progressive increase of X-ray exposure did not lead to significant losses in crystal diffraction. This observation does not necessarily indicate whether the maximum possible hit rate could be achieved and if, for example, the jet consistently recovers to provide diffraction from two crystals on two subsequent pulses. In addition, the observation is of course limited by the resolution of the detector and photon wavelength, which is only 12.2 nm for the PS I 200 reflection. A more thorough test would require a shorter wavelength. Fig. 8 ▸ shows the virtual powder rings formed by summing many background-corrected diffraction patterns, and the resolutions of the four accessible Bragg reflections are shown in Table 1 ▸.

## 6. Conclusions   

By conducting serial diffraction experiments with a high-repetition-rate soft-X-ray free-electron laser, we determined that some of the requirements of SFX experiments at megahertz repetition rates can be realized. Our results suggest that it is feasible to collect crystallography data at pulse repetition rates of up to 4.5 MHz. This was achieved by designing and manufacturing nozzles that produce jets of small diameter and high velocity, which we determined to be the critical parameters for high-rate X-ray FEL diffraction experiments. In the work presented here, velocities of >80 m s^−1^ were achieved with nozzles that can be fabricated reproducibly using components made by ceramic injection molding. Optical images of jets interacting with X-ray pulses at doses up to 1.4 GGy show that stable jets can be maintained when illuminated with X-ray FEL pulse trains consisting of hundreds of pulses at megahertz repetition rates as well as with pulses separated by as little as 221.5 ns (4.5 MHz). Furthermore, we have successfully conducted serial diffraction experiments with megahertz pulse trains on sensitive membrane protein crystals (photosystem I). To the low resolution of Bragg peaks observable in the experiments, the putative pressure wave generated by the X-ray interaction with the jet did not disturb the crystal lattice. As a result of the low photon energy used here, it is not possible to make conclusive remarks regarding changes in the crystal lattice at high resolution. For this, high-resolution studies at the European XFEL with low-viscosity liquid jets are required.

## Figures and Tables

**Table 1:** Resolution for the peaks detected during this experiment

Reflection	Resolution (nm)
〈100〉	24.3
〈110〉	14.0
〈101〉	13.7
〈200〉	12.2
